# Dr Google Is Here to Stay but Health Care Professionals Are Still Valued: An Analysis of Health Care Consumers’ Internet Navigation Support Preferences

**DOI:** 10.2196/jmir.7489

**Published:** 2017-06-14

**Authors:** Kenneth Lee, Kreshnik Hoti, Jeffery David Hughes, Lynne Emmerton

**Affiliations:** ^1^ Division of Pharmacy School of Medicine University of Tasmania Hobart Australia; ^2^ School of Pharmacy Faculty of Health Sciences Curtin University Perth Australia; ^3^ Division of Pharmacy Faculty of Medicine University of Prishtina Prishtina Albania

**Keywords:** health care, information seeking behavior, Internet, chronic disease, patients, surveys

## Abstract

**Background:**

The Internet offers great opportunities for consumers to be informed about their health. However, concerns have been raised regarding its impact on the traditional health consumer-health professional relationship. Our recent survey of 400 Australian adults identified that over half of consumers required some form of navigational support in locating appropriate Web-based health information. We propose that support provided by health professionals would be preferred by consumers; this preference is regardless of whether consumers have a need for navigational support. Secondary analysis of the survey dataset is presented here to quantify consumer-reported support preferences and barriers when navigating Web-based health information.

**Objective:**

We aimed to quantitatively identify consumers’ support preferences for locating Web-based health information and their barriers when navigating Web-based health information. We also aimed to compare such preferences and barriers between consumers identified as needing and not needing support when locating Web-based health information.

**Methods:**

Chi-square (χ^2^) tests identified whether each listed support preference differed between subgroups of consumers classified as needing (n=205, 51.3%) or not needing (n=195, 48.8%) navigational support; degree of association, via phi coefficient (φ) tests, were also considered to ascertain the likely practical significance of any differences. This was repeated for each listed barrier. Free-text responses regarding additional support preferences were descriptively analyzed and compared with the quantitative findings to provide a richer understanding of desired support for health information searches.

**Results:**

Of the 400 respondents, the most preferred mode of navigational support was involvement of health professionals; this was reported by participants identified as needing and not needing navigational support. While there was a significant difference between groups, the degree of association was small (χ^2^_1_ [N=400]=13.2; *P*<.001; φ=.18). Qualitative data from the free-text responses supported consumers’ desire for health professional involvement. The two most commonly reported barriers when navigating desired Web-based health information were (1) volume of available information and (2) inconsistency of information between sources; these were reported by participants with and without a need for navigational support. While participants identified with a need for navigational support were more likely to report volume (χ^2^_1_ [N=387]= 4.40; *P*=.04; φ=.11) and inconsistency of information (χ^2^_1_ [N=387]= 16.10, *P*<.001, φ=.20) as barriers, the degrees of association were small to moderate.

**Conclusions:**

Despite concerns in the literature that the popularity of the Internet could compromise the health consumer-health professional relationship, our findings suggest the contrary. Our findings showed that health professionals were found to be the most commonly preferred mode of navigational support, even among consumers classified as not needing navigational support. Further research into how health professionals could assist consumers with Web-based health information seeking could strengthen the health consumer-health professional relationship amidst the growing use of “Dr Google.”

## Introduction

The relationships between health care consumers and health care professionals have changed over recent decades [1-4]. From the traditional one-way information transfer approach [4], education and communication between health care consumers and health care professionals is now a two-way exchange [2-4]. Similarly, health care consumer autonomy and sovereignty are arguably on the rise [5,6]. In this era, the role of health information in facilitating consumers’ health care roles appears to be of paramount importance [5], particularly given the increasing prevalence of chronic health conditions [7] that require day-to-day self-management [3].

The Internet provides consumers with an avenue to source, query, and publicly debate health information, as well as to self-diagnose and self-manage medical conditions. Concerns have been raised among health care professionals about the potentially negative impact of Web-based health information on the relationships between health care consumers and health care professionals [6,8]. Specifically, some health care professionals are concerned that consumers may make decisions about their health based on misleading or poor-quality information, overriding health care professionals’ recommendations [8,9]. By contrast, a recent study by Laugesen et al found that Web-based health information does not significantly influence treatment concordance, provided there is a good relationship between health care consumers and health care professionals [10]. While a larger body of evidence suggests that health care professionals are, overall, positive about the influence of the Internet on their relationships with health care consumers [8,9,11-14], little research explores the position of health care professionals in this evolving relationship.

Apart from concerns about the influence of the Internet on health care, the issues of variable quality and comprehensibility of available Web-based health information need to be addressed [8,15]; despite these issues, the Internet remains a popular channel for health information [16,17]. Collectively, these factors mean that consumers need to be able to find and appropriately use health information for their health management [18].

Appropriate searching and utilization of Web-based health information requires adequate health literacy [19], as well as digital or computer literacy and media literacy, collectively known as eHealth literacy [20]. While it has been established that the prevalence of limited health literacy [18,21-23] leads to poorer health outcomes and higher rates of hospital admissions and mortality [24,25], far fewer studies have investigated the prevalence of and health outcomes associated with eHealth literacy levels. For example, only one study appears to have specifically explored the prevalence of limited eHealth literacy [26], and one recent study explored associations between eHealth literacy and perceived health status and self-management skills [27]. Furthermore, little has been attempted in terms of interventions that can assist consumers in acquiring quality Web-based health information [28].

In order to inform tailored interventions to assist consumers with acquiring quality and relevant Web-based health information, our earlier research qualitatively explored consumers’ Web-based health information-seeking behaviors and the range of preferences for support with navigating Web-based health information [29]. From the manifest-level themes [30] identified in our qualitative study [29], we developed a questionnaire to survey a representative sample of 400 Australian adults living with chronic health conditions. The survey estimated that approximately half (51.3%, with a precision of 4.9% either side of the true population value) of Australian adult consumers using Web-based health information and living with chronic health conditions require support with navigating Web-based health information [31]; this survey also found that a need for navigational support was more likely among consumers with lower reported levels of eHealth literacy [31].

This study builds on our qualitative study [29] and previous survey [31] by (1) quantitatively identifying key consumer-reported navigational support preferences, (2) quantitatively identifying consumer-reported barriers to navigating desired Web-based health information, and (3) comparing findings from Objectives 1 and 2 between respondents classified as needing or not needing navigational support.

This study also aims to qualitatively explore additional navigational support preferences to provide a richer understanding of desired support for health information searches.

## Methods

### Overview

This study is a secondary analysis of the raw dataset from our previous survey [31], which used a Web-based questionnaire to identify health care consumers’ Web-based health information-seeking behaviors, eHealth literacy, and motivation to engage in their health care (activation). Examples of the types of questions asked in the aforementioned survey pertained to types of health information sought (eg, information about treatment options, medical conditions, diets, and exercises), why health information is sought on the Internet, what actions are taken once Web-based health information has been obtained, all 8 items from the eHealth Literacy scale developed by Norman and Skinner [20], all 13 items from the Patient Activation Measure developed by Hibbard et al [32], usual reason(s) for encountering difficulty with finding pertinent health information on the Internet, and preferred modes of support to assist with obtaining pertinent Web-based health information. The specific provider of the Web-based questionnaire platform used for the aforementioned survey research is Qualtrics, a company that also offers recruitment services via a third-party global research company, Research Now. While our previous analysis detailed an algorithm to identify the proportion of consumers with and without navigational needs when searching for Web-based health information, the secondary analysis reported here provides the first-known insight into navigational support preferences and barriers when finding the desired information and compares these findings between respondents in need of and those not in need of navigational support.

Ethical approval for the research, including the current analyses, was granted by the Curtin University Human Research Ethics Committee (HR06/2013).

### Participants, Recruitment, and Sample Size

The target population of our previous survey study [31] was adults in Australia with a chronic health condition, who had sought health information for their condition on the Internet. Recruitment was contracted by Qualtrics to Research Now to fulfil the quota of 400 submitted questionnaires with a representative sample of Australian adults with the following criteria: ability to easily read and write in English, aged 18 years or older, diagnosis of at least one chronic health condition, and experience with the Internet to find information about their health conditions. These participants were chosen by Research Now from a combination of global Internet surveys or opinion panels, based on the participants’ profiles and whether they would likely meet our stated eligibility criteria [33]. Screening questions that reflect our eligibility criteria were included in the questionnaire as an additional step to ensure that participants were indeed eligible to participate. The sample size of 400 participants was calculated using conservative parameters for prevalence studies such that there was 95% confidence that this study’s reported values would fall within 4.9% on either side of the true population values [34].

### Navigational Needs and Navigational Support Preferences

As defined in our previous survey, “navigational needs” refers to “individuals who report having difficulty finding, and would like support in locating, desired Web-based health information” [31]. This was operationally defined as participants who indicated (1) at least some level of difficulty in finding Web-based health information and (2) a desire for help to find Web-based health information [31]. Building on our definition of navigational needs, this study defines navigational support preferences as the mode by which consumers would prefer support in locating desired Web-based health information.

### Analysis

Statistical analyses were conducted using IBM SPSS version 23. Descriptive statistics were used to identify preferred navigational modes of support (Objective 1) and the most commonly-reported barriers when navigating desired Web-based health information (Objective 2). Chi-square tests of independence (χ^2^) were utilized to identify whether each support preference and barrier differed among the groups of consumers identified with and without a need for navigational support, with phi (φ)-coefficient tests of degrees of association to indicate the potential practical significance of these differences (Objective 3) [35]. Free-text responses regarding additional support preferences were also descriptively analyzed and compared with quantitative results to provide a richer understanding of support preferences.

## Results

### Overview

As described in our previous paper [31], 400 participants completed the questionnaire, and 51.3% (205/400) of these participants were identified with navigational needs. Specifically, “a total of 1104 individuals were invited by Research Now from their diverse participant pool. Consent was obtained from 93.03% (1027/1104) of these participants. Of these 1027 individuals, 50.05% (514/1027) met our eligibility criteria, and 77.82% (400/1027) completed the questionnaire” [31].

### Navigational Support Preferences

In terms of determining participants’ navigational support preferences, participants were asked, “Which of the following ideas would help you find health-related information that you need?” The most commonly reported mode of support, by both participants with and without navigational needs, was health care professionals (Table 1). While all reported modes of support (apart from the “other” option) were significantly more likely to be reported by participants with navigational needs, the φ coefficients suggest only a small degree of association.

**Table 1:** Support preferences for navigating Web-based health information (N=400).

Modes of support	No navigational needs, n (%^a,b^)	Navigational needs, n (%^a,c^)	Total, n (%^a^)
Health care professionals to recommend websites (χ^2^_1_ [N=400]=13.2; *P*<.001; φ=.18)	112 (57.4)	153 (74.6)	265 (66.3)
An icon on each website to indicate whether it is from a trustworthy source (χ^2^_1_ [N=400]=8.8; *P*=.003; φ=.15)	96 (49.2)	131 (63.9)	227 (56.8)
Blocking of unreliable websites (χ^2^_1_ [N=400]=6.8; *P*=.01; φ=.13)	82 (42.1)	113 (55.1)	195 (48.8)
Improvement in the layout of websites (χ^2^_1_ [N=400]=12.8; *P*<.001; φ=.18)	35 (17.9)	69 (33.7)	104 (26.0)
Workshops on how to find trustworthy health-related information on the Internet (χ^2^_1_ [N=400]=3.9; *P*=.049; φ=.10)	17 (8.7)	31 (15.1)	48 (12.0)
Other	9 (4.6)	4 (2.0)	13 (3.3)

^a^Respondents could select multiple options (percentages do not total 100%).

^b^Percentages are based on the 195 participants with no navigational needs.

^c^Percentages are based the 205 participants with navigational needs.

Elaborating on the “other” option, the majority of additional modes of support suggested by participants reiterate and elaborate on one or more of the response options listed in Table 1. The below comment by one of the participants illustrates the desire for health care professionals to play a role in supporting Web-based health information seeking:

...maybe a handout of trusted web site addresses (specific to the condition discussed) could be given to the patient at the time of the visit to the doctor? This would make it easy for the patient to find out more specific information regarding their condition and know that the information they are reading is trustworthy and relevant.

ID132

Participants also suggested that a centralized health portal or database containing relevant and quality health information could be beneficial:

A large, centrally managed, government endorsed database of conditions, treatments, side effects and associated forums, institutes/foundations might be helpful. Unbiased, Australian, featuring advice from eminent specialists, and importantly, featured stories showing perspectives from sufferers on how they manage their conditions. I still find myself having to get my information from American sites quite often.

ID228

A few participants expressed a desire for medically-related search engines. One participant (ID197) said, “Google should have a doctor’s section (where you can read everything that is medically related).” Another participant (ID198) posed the question, “Search engine purely for medical research?”

Other participants expressed a desire for greater online communicative features. As one participant (ID171) expressed it, “Online chat services, just like hotline.” Another participant (ID236) felt that a “Website comprised of common information and online chat function” would be helpful.

Although few participants quantitatively indicated that they would welcome workshops to help them find health information on the Internet (Table 1), some indicated a desire to be better educated on how to use Web-based health information effectively. A notable example was this:

With the ability for anyone to put any information on the internet i [sic] think there is a lot of ill-informed information on the internet. It really depends on what your beliefs are about health as to what information is useful. I think there should be more information on what people should be asking their doctor or other health professionals in order to get the best information for them. Perhaps more sites on checklists on things to consider with your health and sources of information, what to ask, alternative options for health information.

ID296

Participant ID296’s response also suggests the need to be educated on how to have more productive interactions with health care professionals.

### Perceived Barriers to Navigating Web-Based Health Information

Of the 400 participants, only 13/400 (3.3%) indicated that they never experienced difficulty finding relevant health information on the Internet. Table 2 summarizes the potential barriers to navigating Web-based health information, as reported by the remaining 387 participants. These 387 participants were asked, “When you do have difficulty finding helpful health-related information online, what is/are the usual reason(s)?” Overall, perceived barriers were similarly reported by both participants with and without navigational needs, except for one finding: the navigational needs group was moderately associated with a barrier relating to conflicting information obtained from different websites. Additionally, the two most commonly reported barriers by both groups were the volume of available information and conflicting information obtained from different websites.

**Table 2:** Perceived barriers to navigating Web-based health information (N=387).

Perceived barriers	No navigational needs, n (%^a,b^)	Navigational needs, n (%^a,c^)	Total, n (%^a^)
There is a large number of websites available on the Internet (χ^2^_1_ [N=387]=4.4; *P*=.04; φ=.11)	80 (44.0)	112 (54.6)	192 (49.6)
Information obtained from different websites sometimes does not match up (χ^2^_1_[N=387]=16.1; *P*<.001; φ=.20)	65 (35.7)	115 (56.1)	180 (46.5)
The information I come across contains a lot of medical terms or jargon.	55 (30.2)	77 (37.6)	132 (34.1)
I do not know what I am looking for.	24 (13.2)	37 (18.0)	61 (15.8)
There appears to be a lack of information specific to my needs.	18 (9.9)	25 (12.2)	43 (11.1)
Other	27 (14.8)	11 (5.4)	38 (9.8)

^a^Respondents could select multiple options (percentages do not total 100%).

^b^Percentages are based on the 182 participants with no navigational needs.

^c^Percentages are based on the 205 participants with navigational needs.

## Discussion

### Principal Findings

Health care professionals were reported to be the most commonly selected option to assist health care consumers in finding credible information on the Internet. Specifically, participants indicated a desire for health care professionals to recommend suitable websites. Amidst some health care professionals’ concerns that consumers’ use of the Internet for health information could negatively impact the relationships between health care consumers and health care professionals [8,9], this study suggests that these concerns may be unfounded, at least among people with a chronic disease. The findings of this research support a study by Laugesen et al on the impact of Web-based health information on patients’ treatment compliance [10].

A desire for health care professionals’ support was significantly more likely to be reported by participants with navigational needs. Given that our previous study [31] found that participants with lower reported eHealth literacy levels were more likely to have navigational needs, this study suggests that health care professionals can have a role to play in supporting health information navigation on the Internet, particularly among consumers with lower levels of eHealth literacy. Nevertheless, the small degree of association between participants with and without navigational needs and their desire for health care professionals’ support suggests that most lay consumers would be amenable to support from their health care professionals when navigating the Internet. Thus, this study provides reassurance that health care professionals are valued in an age where the Internet is a popular channel for obtaining health information [17]. However, we propose that the relationships between health care consumers and health care professionals would benefit from health care professionals acknowledging to their patients that Web-based information can be found and used effectively and that health care professionals can provide guidance as required. This time investment may indeed strengthen their relationship with health care consumers and assist in the eHealth literacy of their patients. However, further investigation would be required to determine the specific roles health care professionals could play in guiding lay consumers to become better informed about their health.

This study identified a number of perceived barriers when navigating Web-based health information. Specifically, the volume of available information on the Internet and the inconsistency of information obtained from different sources were identified as the two most commonly reported barriers across consumers with and without navigational needs. However, these two barriers were significantly more likely to be reported by participants with navigational needs, suggesting the need for greater efforts to be focused on addressing these barriers among consumers with navigational needs. In particular, a moderate association was found between the lack of consistency in information obtained from different sources among participants with navigational needs; this finding appears logical given the plethora of Web-based health information that can result in confusion and may not be directly comparable or may contain conflicting information or advice [15], hence the need for navigational support for this group. Consumers without navigational needs may be more competent and confident in comparing Web-based information to guide their decisions. Thankfully, as consumers with navigational needs are more likely to demonstrate a desire for navigational support from health care professionals, this study further highlights the important roles health care professionals can play in guiding consumers to Web-based health information.

When compared with previous studies, the overabundance of Web-based health information has indeed been reported as a potential issue with using the Internet as a channel for obtaining health information, as indicated in a review by Cline and Haynes [15]. Some studies suggest that information overload and ambiguity or inconsistency of information (not specific to health) can lead to patient confusion [5,36]. One study applied this concept to health care and found that information overload can influence a patient’s cognition, and ambiguity or inconsistency of health information has both cognitive and affective effects [5]. While this study did not specifically assess information overload, the prevalence of this issue as a barrier suggests the need for initiatives to streamline search results and tailor suggested websites to individuals’ needs and health literacy; attempts to partially address this need appear to have been considered by Google, as evidenced in the implementation of health information in their Knowledge Graphs [37]. In a Knowledge Graph, health information is presented as a summary with information drawn from credible sources, including the Mayo Clinic. Another approach to addressing barriers to navigating Web-based health information is the re-design of website user interfaces to align with consumers’ information-seeking behaviors [38,39]. Overall, findings from this study highlight barriers to navigation commonly reported by consumers, thereby providing a focus for the development of future and current interventions to assist consumers in navigating Web-based health information.

The need to filter information was also recognized by participants who suggested a centralized portal for health information, provision of handouts with a list of trusted websites, and a “Google-esque” search engine that only displays medically-related health information from reputable sources. When compared with the responses listed in Table 1, one of the common themes is a desire for a reduction in information sources. For example, “An icon on each website to indicate whether it is from a trustworthy source” and “Blocking of unreliable websites” were among the most commonly rated modes of support (Table 1); both modes of support, if implemented, would arguably result in more relevant search results and more reliable content. Similarly, one could argue that by having health care professionals recommend websites, information sources searched by consumers could be reduced in volume. Collectively, this study suggests that a key perceived barrier to navigating Web-based health information is the volume of available information and that the preferred modes of support echo the desire to effectively distil available information into a smaller set of pertinent and quality sources.

### Strengths and Limitations

A key strength of this study is the “within-method” triangulation [40] that was used to compare quantitative and free text (qualitative) findings from this study. The complementary nature of the collective findings serves to broaden understanding of the phenomenon and is a tactic to improve internal consistency or reliability of findings. Additionally, cross-validation, also known as “between-method” triangulation [40], was used to compare findings from similar studies with findings from this study.

Our structured, third party–administered questionnaire limited clarification and confirmation of free-text responses with participants. However, the use of a number of free-text responses and within-method data triangulation enabled a richer understanding of the phenomena beyond the understanding that would have been obtained if only quantifiable response options had been used [40].

As this study is a secondary analysis of a raw dataset, the methodological limitations recognized in our earlier paper [31] also apply here. For example, our reference to “navigation” only referred to finding (searching for or sourcing) Web-based health information. The findings of this study may not extend to consumers’ preferences for assistance in interpreting and applying health information. Considering our key finding about the preference for assistance from a health care professional in finding relevant health information, it may be assumed that this type of “health coach” role would extend to assisting the health care consumer to manage the results of Internet searches, and specifically, understanding the relevance of information and how it may be applied at an individual level. Additionally, characteristics of the sample of participants, while reported by the third-party provider (Research Now) to be representative of our target population, cannot be confirmed. As such, the transferability of our findings to the wider population cannot be assured. However, given the moderately large sample size and the congruency between the quantitative and free-text responses of our participants, findings from this study may be applicable at least to the broader population of health information consumers living with chronic health conditions.

### Further Research

The findings of this study suggest that future studies exploring consumers’ health information-seeking behaviors should explore the concept of information overload and its effects on individuals having different levels of eHealth literacy. The concept of information overload, while investigated at length in other disciplines such as management [41], does not appear to be widely studied in the context of Web-based health information.

The likely acceptability of assistance from health care professionals is also worthy of further investigation. It is unclear whether health care professionals feel sufficiently skilled and confident to inform consumers about suitable information sources on the Internet for specific health conditions. Further research could explore health care professionals’ ability to assess consumers’ levels of eHealth literacy and subsequently direct them to suitable, reliable Internet sources. Given the increasing popularity of mobile-based health care and the advent of mobile apps [42], further research could also examine whether there is a need for consumers to be directed to appropriate apps and whether health care professionals are able to provide appropriate recommendations on suitable consumer health apps. To our knowledge, there has been no research into how health care professionals might integrate this type of assessment and advice into regular health consultations.

Regardless of how health care professionals might assist consumers in navigating Web-based health information, it is important to recognize that consumers’ preferences for seeking or obtaining health information vary, and this difference can influence health behaviors. For example, a study by Williams-Piehota et al found that female participants with a lower desire for seeking health information were less likely to participate in a mammography after being given detailed information about mammograms as compared with when they were given concise information [43]. Conversely, female participants with a higher desire for seeking health information were less likely to participate in a mammography after being given concise information as compared with detailed information [43]. Thus, future interventions need to be cognizant of information-seeking needs at the individual level, to support desirable health behaviors.

### Conclusions

This study explored consumer-perceived barriers to navigating desired Web-based health information and consumers’ navigational support preferences. Our findings identified that the volume of available information and the inconsistency of information obtained from different information sources were the most commonly identified barriers. Despite concerns that the Internet and consumer sovereignty could negatively impact the relationships between health care consumers and health care professionals, our findings suggest the contrary, as health care professionals were reported as the most commonly selected option for providing navigational support. Further exploration of how health care professionals could assist consumers with their Web-based health information-seeking could see the strengthening of this relationship amidst the growing use of the Internet for obtaining health information.

## References

[1] McMullan M (2006). Patients using the internet to obtain health information: how this affects the patient-health professional relationship. Patient Educ Couns.

[2] Ishikawa H, Hashimoto H, Kiuchi T (2013). The evolving concept of “patient-centeredness” in patient-physician communication research. Soc Sci Med.

[3] Bodenheimer T, Lorig K, Holman H, Grumbach K (2002). Patient self-management of chronic disease in primary care. JAMA.

[4] Hoving C, Visser A, Mullen PD, van den Borne B (2010). A history of patient education by health professionals in Europe and North America: from authority to shared decision making education. Patient Educ Couns.

[5] Gebele C, Tscheulin DK, Lindenmeier J, Drevs F, Seemann A (2014). Applying the concept of consumer confusion to healthcare: development and validation of a patient confusion model. Health Serv Manage Res.

[6] Hardey M, Harlow E, Webb SA (2003). Consumers, the internet and the reconfiguration of expertise. Information and Communication Technology in the Welfare Services.

[7] Nugent R (2008). Chronic diseases in developing countries: health and economic burdens. Ann N Y Acad Sci.

[8] Murray E, Lo B, Pollack L, Donelan K, Catania J, Lee K, Zapert K, Turner R (2003). The impact of health information on the internet on health care and the physician-patient relationship: National U.S. survey among 1.050 U.S. physicians. J Med Internet Res.

[9] de Oliveira JF (2014). The effect of the internet on the patient-doctor relationship in a hospital in the city of Sao Paulo. J Inf Syst Technol Manag.

[10] Laugesen J, Hassanein K, Yuan Y (2015). The impact of internet health information on patient compliance: a research model and an empirical study. J Med Internet Res.

[11] Iverson SA, Howard KB, Penney BK (2008). Impact of internet use on health-related behaviors and the patient-physician relationship: a survey-based study and review. J Am Osteopath Assoc.

[12] Hart A, Henwood F, Wyatt S (2004). The role of the Internet in patient-practitioner relationships: findings from a qualitative research study. J Med Internet Res.

[13] Wald HS, Dube CE, Anthony DC (2007). Untangling the Web--the impact of Internet use on health care and the physician-patient relationship. Patient Educ Couns.

[14] Stevenson F, Kerr C, Murray E, Nazareth I (2007). Information from the Internet and the doctor-patient relationship: the patient perspective--a qualitative study. BMC Fam Pract.

[15] Cline RJ, Haynes KM (2001). Consumer health information seeking on the internet: the state of the art. Health Educ Res.

[16] Andreassen HK, Bujnowska-Fedak MM, Chronaki CE, Dumitru RC, Pudule I, Santana S, Voss H, Wynn R (2007). European citizens' use of e-health services: a study of seven countries. BMC Public Health.

[17] Fox S, Duggan M (2013). Pew Research Center.

[18] Baker D, Wolf M, Feinglass J, Thompson J, Gazmararian J, Huang J (2007). Health literacy and mortality among elderly persons. Arch Intern Med.

[19] Nutbeam Don (2009). Defining and measuring health literacy: what can we learn from literacy studies?. Int J Public Health.

[20] Norman C, Skinner H (2006). eHealth literacy: essential skills for consumer health in a networked world. J Med Internet Res.

[21] Manning DM, Dickens C (2006). Health literacy: more choice, but do cancer patients have the skills to decide?. Eur J Cancer Care (Engl).

[22] Australian Bureau of Statistics (2006). ABS.

[23] Kutner M, Greenberg E, Jin Y, Paulsen C (2006). NCES.

[24] Berkman N, Sheridan S, Donahue K, Halpern D, Crotty K (2011). Low health literacy and health outcomes: an updated systematic review. Ann Intern Med.

[25] Bostock S, Steptoe A (2012). Association between low functional health literacy and mortality in older adults: longitudinal cohort study. BMJ.

[26] Xesfingi S, Vozikis A (2016). eHealth literacy: in the quest of the contributing factors. Interact J Med Res.

[27] Neter E, Brainin E (2017). Perceived and performed ehealth literacy: survey and simulated performance test. JMIR Hum Factors.

[28] Lee K, Hoti K, Hughes JD, Emmerton LM (2014). Interventions to assist health consumers to find reliable online health information: a comprehensive review. PLoS One.

[29] Lee K, Hoti K, Hughes JD, Emmerton L (2014). Dr Google and the consumer: a qualitative study exploring the navigational needs and online health information-seeking behaviors of consumers with chronic health conditions. J Med Internet Res.

[30] Boyatzis R (1998). Transforming qualitative information: thematic analysis and code development.

[31] Lee K, Hoti K, Hughes JD, Emmerton LM (2015). Consumer use of “Dr Google”: a survey on health information-seeking behaviors and navigational needs. J Med Internet Res.

[32] Hibbard JH, Mahoney ER, Stockard J, Tusler M (2005). Development and testing of a short form of the patient activation measure. Health Serv Res.

[33] Research Now (2017). Research Now.

[34] National Statistical Service NSS.

[35] Field A (2013). Discovering statistics using SPSS.

[36] Walsh G, Hennig-Thurau T, Mitchell V (2007). Consumer confusion proneness: scale development, validation, and application. J Mark Manag.

[37] Ramaswami P (2015). Googleblog.

[38] Pang PC, Chang S, Verspoor K, Pearce J (2016). Designing health websites based on users' web-based information-seeking behaviors: a mixed-method observational study. J Med Internet Res.

[39] Cui L, Carter R, Zhang G (2014). Evaluation of a novel conjunctive exploratory navigation interface for consumer health information: a crowdsourced comparative study. J Med Internet Res.

[40] Jick TD (1979). Mixing qualitative and quantitative methods: triangulation in action. Admin Sci Q.

[41] Eppler MJ, Mengis J (2004). The concept of information overload: a review of literature from organization science, accounting, marketing, MIS, and related disciplines. Inf Soc.

[42] Santo K, Richtering SS, Chalmers J, Thiagalingam A, Chow CK, Redfern J (2016). Mobile phone apps to improve medication adherence: a systematic stepwise process to identify high-quality apps. JMIR Mhealth Uhealth.

[43] Williams-Piehota P, Pizarro J, Schneider TR, Mowad L, Salovey P (2005). Matching health messages to monitor-blunter coping styles to motivate screening mammography. Health Psychol.

